# Metabolomics Reveal Nanoplastic-Induced Mitochondrial Damage in Human Liver and Lung Cells

**DOI:** 10.1021/acs.est.2c03980

**Published:** 2022-08-25

**Authors:** Siyi Lin, Hongna Zhang, Chen Wang, Xiu-Li Su, Yuanyuan Song, Pengfei Wu, Zhu Yang, Ming-Hung Wong, Zongwei Cai, Chunmiao Zheng

**Affiliations:** † State Key Laboratory of Environmental and Biological Analysis, Department of Chemistry, Hong Kong Baptist University, Hong Kong 999077, China; ‡ State Environmental Protection Key Laboratory of Integrated Surface Water-Groundwater Pollution Control, School of Environmental Science and Engineering, Southern University of Science and Technology, Shenzhen 518055, China; § State Key Laboratory of Environmental Criteria and Risk Assessment, Chinese Research Academy of Environmental Sciences, Beijing 100012, China; ∥ Consortium on Health, Environment, Education and Research (CHEER), Department of Science and Environmental Studies, The Education University of Hong Kong, Hong Kong 999077, China

**Keywords:** plastic particles, cytotoxicity, mitochondria, electron transport chain, energy metabolism

## Abstract

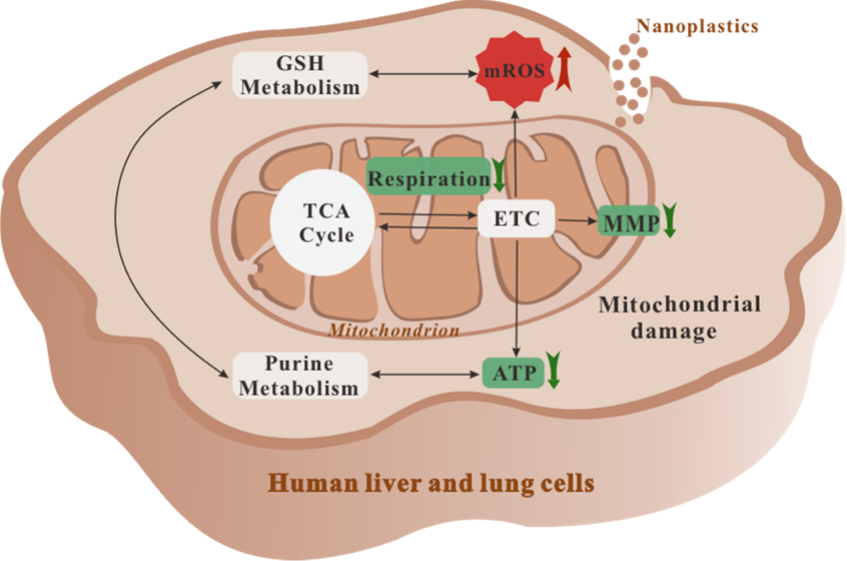

Plastic debris in the global biosphere is an increasing concern, and nanoplastic (NPs) toxicity in humans is far from being understood. Studies have indicated that NPs can affect mitochondria, but the underlying mechanisms remain unclear. The liver and lungs have important metabolic functions and are vulnerable to NP exposure. In this study, we investigated the effects of 80 nm NPs on mitochondrial functions and metabolic pathways in normal human hepatic (L02) cells and lung (BEAS-2B) cells. NP exposure did not induce mass cell death; however, transmission electron microscopy analysis showed that the NPs could enter the cells and cause mitochondrial damage, as evidenced by overproduction of mitochondrial reactive oxygen species, alterations in the mitochondrial membrane potential, and suppression of mitochondrial respiration. These alterations were observed at NP concentrations as low as 0.0125 mg/mL, which might be comparable to the environmental levels. Nontarget metabolomics confirmed that the most significantly impacted processes were mitochondrial-related. The metabolic function of L02 cells was more vulnerable to NP exposure than that of BEAS-2B cells, especially at low NP concentrations. This study identifies NP-induced mitochondrial dysfunction and metabolic toxicity pathways in target human cells, providing insight into the possibility of adverse outcomes in human health.

## Abstract 2

Metabolomics clarified the mechanisms of nanoplastic-induced mitochondrial damage in human cells.

## Introduction

Plastic particles with a diameter of less than 0.1 μm (nanoplastics (NPs)) have been extensively manufactured and used as engineered nanomaterials in various products.^1^ In particular, NPs are used in numerous consumer and personal care products, such as microbead-containing shampoos and scrubs.^2^ Furthermore, human activities have resulted in the discharge of large amounts of plastic waste (∼99 million tons),^3^ which can continually release secondary NPs during degradation under the action of ultraviolet radiation, hydrolytic processes, mechanical abrasion,^4,5^ and biological processes.^6,7^ The widespread application and secondary production of NPs have resulted in environmental contamination with a measurable concentration of 0.04 mg/mL in the aquatic environment.^8^ The accumulation of plastic particles in the organs of marine life has been well documented,^9,10^ and these particles can bioaccumulate in higher trophic biological species via the food chain,^11−13^ which may ultimately lead to environmental exposure in humans. However, NP-related health impacts on humans are not well understood.

Humans routinely ingest NPs from plastic-contaminated food,^14^ which is one of the primary routes of NP exposure. NPs with a diameter less than 100 nm are of particular importance because they can cross cell membranes into the lymph and blood circulation and accumulate in various tissues and organs.^15^ These NPs inevitably undergo metabolic processes in the liver.^16^ Inhalation is another critical NP exposure pathway in humans as NPs suspended in air can be directly inhaled into the respiratory system and penetrate deep into the lungs.^14,15^Associations between NP exposure and inflammation, immunotoxicity, and neurological dysfunction have been demonstrated in mice.^17−19^*In vivo* studies have found that NPs are distributed in multiple organs (e.g., liver and lungs).^19,20^ Furthermore, *in vitro* data have shown that the internalization of NPs occurs in various human cell lines,^21,22^ indicating that damage to sensitive organelles may be a primary mechanism of NP-induced toxicity in cells. The endoplasmic reticulum (ER) and mitochondrion are crucial organelles and are functionally coordinated, and their interaction regulates many intracellular processes.^23^ ER stress-related metabolic changes have been observed after NP exposure in human lung cells, indicating that mitochondrial function is a potential target of NPs.^24^ Because the lung and liver are two of the organs most likely to be directly exposed to NPs, there is a need to improve our understanding of the metabolic mechanisms of NP toxicity, such as how NPs interact with organelles and the associated downstream metabolic effects.

In the current toxicological paradigm, biological response pathways could lead to toxicity pathways and ultimately cause adverse health outcomes.^25^ As such, predisease events that occur at the molecular and cellular levels can be used to predict the outcomes of exposure to environmental pollutants.^26^ High-throughput mass spectrometry (MS)-based metabolomics profiling can be used to comprehensively and systematically analyze important small-molecule endogenous metabolites within biological systems.^27^ In addition, cumulative metabolic changes in response to external stress can indicate the physiological state of a cell,^28^ and metabolites can act as phenotype modulators. The liver and lung are highly involved in the metabolism and contain the most well-established and complete metabolic enzymes (e.g., mixed-functional oxidase) in the body,^29^ while the NP-induced cellular responses of endogenous metabolites in the two organs have not been well studied and thus require indepth investigation. Therefore, it is necessary to evaluate the toxicity of environmental exposure to NPs and the associated metabolic pathways at the cellular level.

This study explored NP-induced toxic effects in the mitochondria and the related metabolic pathways in two normal human cell lines: a liver (hepatic) cell line (L02 cells) and a lung epithelial cell line (BEAS-2B cells). These cell lines have been broadly used in environmental toxicological studies to reveal the impacts of pollutant-induced liver/lung-specific metabolic functions.^30,31^ A low-concentration exposure group was designed according to the concentrations found in the aquatic environment (0.04 mg/mL).^8^ Mitochondrial damage was demonstrated by overproduction of mitochondrial reactive oxygen species (mROS) and suppression of the mitochondrial membrane potential (MMP) and mitochondrial respiration. Such changes are essential precursors to adverse outcomes. Nontarget metabolomics was further used to confirm that mitochondria are the organelles most vulnerable to NP exposure and investigated metabolic changes. This study provides new insights into NP-induced toxicity pathways and the underlying toxicity mechanisms in human cell lines.

## Materials and Methods

### Nanoplastics and Cell Culture

Nonfluorescent and fluorescent polystyrene 80 nm NP suspensions (free of sodium azide; 2.5%, w/v; density: 1.064 g/cm^3^) were purchased from BaseLine ChromTech Research Centre (Tianjin, China). The nonfluorescent NPs were analyzed by transmission electron microscopy (TEM, FEI Talos F200X, Thermo Fisher) (Figure S1). The normal human hepatic L02 cell line (Shanghai Cell Bank of Type Culture Collection of the Chinese Academy of Sciences) and the normal human lung epithelial BEAS-2B cell line (ATCC, CRL-9609) were used in this study. L02 cells were cultured in high-glucose Dulbecco’s modified Eagle’s medium (DMEM) supplemented with 10% fetal bovine serum (FBS) and 1% penicillin–streptomycin (Gibco, Thermo Fisher Scientific, Waltham, MA). BEAS-2B cells were cultured in bronchial epithelial cell growth basal medium supplemented with bronchial epithelial cell growth medium (BEGM) SingleQuots supplements, growth factors, and MycoZap Plus-CL (Lonza, Walkersville, MD). The two cell lines were maintained at 37 °C in a 95:5 air/CO_2_ humid atmosphere.

### Cell Viability Assay

L02 and BEAS-2B cells were seeded into 96-well plates at a density of 2 × 10^4^ cells/well and cultured for 24 h. The L02 cells were cultured in DMEM containing 1% penicillin–streptomycin, and the BEAS-2B cells were cultured in BEGM. After culturing, the cells were treated with 0, 0.006, 0.0125, 0.03125, 0.0625, 0.125, or 0.25 mg/mL NPs that had been dissolved in phosphate-buffered saline (PBS; 5%, v/v) (Gibco, Thermo) and then exposed for 48 h. Six replicates of each concentration were used. The intermediate exposure concentration (0.03125 mg/mL) was designed according to the reported NP concentration in the aquatic environment (0.04 mg/mL).^8^ The lower concentrations were designed with a 2-fold concentration gradient of the reported concentration in the aquatic environment, while higher concentrations were used to explore mechanisms of toxicity. It is essential to point out that NPs in the actual environment are complex with a wide range of sizes and shapes; therefore, the reference concentration of 0.04 mg/mL has limitations. However, in the face of a lack of data, this value was used. Before each treatment, the stock suspensions of NPs were homogenized by ultrasonication in a water bath (30–40 kHz) for more than 30 min and then vortexed for 1–3 min to destroy aggregates. After NP exposure, cell viability was assessed using a commercial Cell Counting Kit-8 (CCK-8) assay (Dojindo, Japan). Wells containing NPs, CCK-8 reagent, and complete medium were used as controls. After these treatments, the absorbance of cells at 490 nm was detected on a Victor X3 Multilabel Plate Reader (PerkinElmer, Waltham, MA).

### NP Cellular Internalization

NP internalization by L02 and BEAS-2B cells was examined by fluorescent labeling and TEM. For fluorescent labeling, the two cell lines were seeded into six-well glass-bottom plates (Cellvis), incubated overnight, and then treated with 0, 0.006, 0.0125, 0.03125, 0.0625, 0.125, or 0.25 mg/mL fluorescent NPs for 1 h.^32^ The cells were then fixed with 4% paraformaldehyde (PFA) for 20 min. The fixed cells were then washed with Tris-buffered saline containing 0.1% Tween-20 (TBS-T) for 10 min and stained with 4′,6-diamidino-2-phenylindole (DAPI, NucBlue Fixed Cell Reagent Probes Reagent, Thermo) for 20 min. After staining, the cells were washed with PBS three times for 5 min each and then imaged using confocal laser scanning microscopy (CLSM) (Nikon C2si Plus). The excitation and emission maxima of the NPs were 488 and 518 nm, respectively, and those of DAPI were 360 and 460 nm, respectively.

For TEM analysis, the cells were first cultured in 10 cm dishes overnight. The following day, they were exposed to nonfluorescent NP treatment for 48 h. Both cell types were exposed to a low-NP concentration (0.0125 mg/mL), while the high concentrations were 0.125 mg/mL for L02 cells and 0.25 mg/mL for BEAS-2B cells. After exposure, the cells were washed with PBS, fixed with 2.5% glutaraldehyde (30 min, 4 °C), transferred to 1.5 mL Eppendorf tubes, and centrifuged at 15 000*g* for 3–5 min depending on the cell type. The supernatants were then removed, and 1 mL of fresh glutaraldehyde fixative solution was slowly added along the tube wall. The cells were then stored at 4 °C overnight for the following treatments. Further details are described in the Supporting Information.

### mROS and MMP Determination

L02 and BEAS-2B cells were seeded into six-well glass-bottom plates and treated with NPs at the same concentrations as those used in the TEM observations (L02 with 0, 0.0125, and 0.125 mg/mL; BEAS-2B with 0, 0.0125, and 0.25 mg/mL). The medium was removed after exposure, and 5 μM MitoSOX Red (M3600, Thermo) was added to each well. The cells were then incubated for 10 min, subsequently washed with PBS, and then fixed with 4% PFA for 20 min. The MMP was determined using a 5,5′,6,6′-tetrachloro-1,1′,3,3′-tetra-ethylbenzimidazolocarbo-cyanine iodide (JC-1) assay (Beyotime Institute of Biotechnology, Beijing, China) according to the manufacturer’s instructions. An equal volume (1 mL) of 5 μg/mL JC-1 staining solution was added to the cells that were then incubated for 20 min and subsequently washed with PBS. Carbonyl cyanide *m*-chlorophenyl hydrazone was used as a positive control. The presence of mROS and MMP in cells was observed by CLSM. The excitation and emission wavelengths were as follows: MitoSOX Red, 510 and 580 nm; mitochondrial JC-1 monomers, ∼490 and 530 nm; and JC-1 aggregates, ∼525 and 590 nm, respectively.

### Mitochondrial Stress Test

The Agilent Seahorse Mito Stress Test Kit was used to assess mitochondrial respiration. L02 and BEAS-2B cells were seeded into eight-well assay miniplates at a density of 2 × 10^4^ cells/well. The exposure concentrations were the same as those used for mROS and MMP determination. After 48 h of exposure, the culture medium was removed and replaced with Agilent Seahorse XF Assay Medium (containing 10 mM glucose, 1 mM sodium pyruvate, and 2 mM l-glutamine). Determinations were carried out according to the manufacturer’s instructions, and the details are provided in the Supporting Information.

### Nontarget Metabolomics Screening

The two cell lines (density: 1 × 10^6^ cells/well) were seeded into 6 cm dishes and treated with NPs for 48 h at the same concentrations as those used for mROS and MMP determinations. Twelve replicates were analyzed for each group: nine of these replicates were prepared for nontarget metabolomics, and three of these replicates were used to measure the total protein concentrations for normalization. The procedures have been described in our previous studies.^31,33^ After 48 h of NP exposure, the samples for the metabolomics study were quickly rinsed twice with PBS, quenched with 400 μL of chilled methanol (MeOH)/H_2_O (4:1, v/v) containing 1 μg/mL 4-chloro-phenylalanine as the first internal standard. The cell samples were then disrupted by five freeze–thaw cycles in liquid nitrogen and then centrifuged at 15 000*g* (10 min, 4 °C). Their supernatants were then collected and subsequently dried at 4 °C in a Max-Up IR vacuum concentrator (NB-504CIR, N-Biotek Inc., GyeongGi-Do, Korea). The residues were resuspended in MeOH/H_2_O (1:1, v/v) containing 0.5 μg/mL glutamine-^13^C_5_ as the second internal standard. The solvent quantities used were proportional to the total cellular protein concentrations that were previously measured. The samples were then analyzed by a Thermo Scientific ultrahigh-performance liquid chromatography system coupled to a Q-Exactive Focus Hybrid Quadrupole-Orbitrap mass spectrometer (QE Orbitrap MS) in both positive and negative modes. A quality control sample was prepared containing a mixture of all samples. The instrument conditions are presented in Table S1.

### Biological Determinations Related to Metabolic Pathways

To further explore the mechanisms of NP-induced cytotoxicity, cellular ATP and xanthine oxidase (XOD) concentrations were measured. Cells were seeded into six-well plates at a density of 3 × 10^5^ cells/well, incubated overnight, and treated with NPs for 48 h at the same concentrations described in the nontarget metabolomics protocols. An ATP Assay Kit (Beyotime Institute of Biotechnology) and an XOD Assay Kit (Nanjing Jiancheng Bioengineering Institute) were used to measure the concentrations of ATP and XOD according to the manufacturers’ instructions. The measurements were normalized to the total protein concentrations.

### Nontarget Metabolomics Data Processing

The data analysis was conducted as in our previous studies.^31,33,34^ The data were acquired and pretreated using Xcalibur v4.1 software (Thermo). For peak detection and alignment, the metabolic features in the global metabolomics data were extracted by the XCMS package (version 1.46.0) using R.^35^ This afforded a list of data (mass-to-charge ratio, retention time, and peak intensity) in CSV format. The metabolic features of quality control samples with a standard deviation greater than 30% relative and disturbed signals in the blanks were excluded to eliminate interference (e.g., false positives that occurred because of instrumental fluctuations). The remaining qualified data were analyzed by volcano plots and partial least squares with discriminant analysis (PLS-DA) using SIMCA-P v13 (Umetrics, Umea, Sweden). Metabolic features that differentiated the control group from the NP-treated groups were further identified based on (1) a *p* value < 0.05 and (2) a fold change greater than 1.2 or less than 0.8. The MS/MS data were further processed using Compound Discovery software (Thermo Scientific) for metabolite identification, which involved matching the retention time, accurate precursor mass, isotope pattern, and MS/MS spectra of the metabolites to those of authentic standards from metabolite libraries. The metabolite libraries used were the mzCloud library, the open-access metabolic databases of the Human Metabolome Database (https://hmdb.ca/), and METLIN (http://metlin.scripps.edu/). The molecular weight tolerance was set at ±10 ppm with respect to the theoretical values for each metabolite. Metabolic pathways were analyzed by MetaboAnalyst 5.0 (https://www.metaboanalyst.ca/) based on the Kyoto Encyclopedia of Genes and Genomes (KEGG) database.^36^

### Statistical Analysis

All descriptive data were analyzed using SPSS software v26.0 (IBM, Chicago, IL) and MATLAB (R2021a). The results are reported as the mean ± standard deviation. Statistical comparisons of groups were performed by one-way analysis of variance with Dunnett’s T3 or Bonferroni post hoc tests. Statistical significance was indicated by *p* < 0.05.

## Results and Discussion

### Cell Viability Assay and Internalization

**Figure 1:**
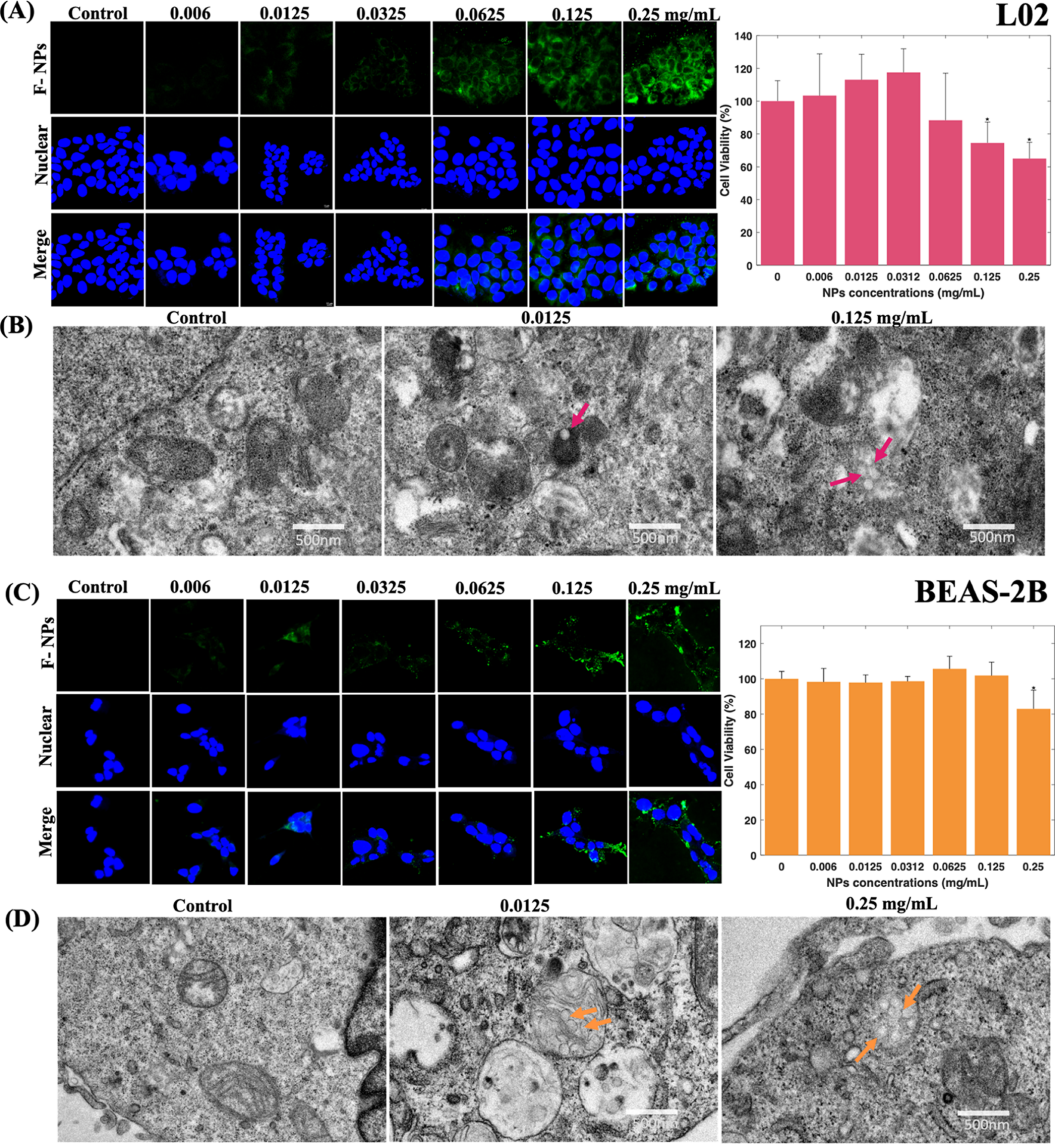
Uptake of 80 nm NPs by normal human hepatic L02 and lung BEAS-2B cell lines and their cell viability at a series of concentrations: the internalization of fluorescent NPs (F-NPs) (A, C) and nonfluorescent NPs via TEM analysis (B,D). Data of cell viability are presented as the mean ± standard deviation of *n* = 6, *p < 0.05.

The L02 cell viability slightly increased in the low-NP-concentration groups (0.006, 0.0125, 0.0312 mg/mL) and significantly decreased in the high-NP-concentration groups (0.125 and 0.25 mg/mL) compared with the control (Figure 1A,C). The increase in viability indicated that the L02 cells were stimulated to detoxify the NPs as a survival response to an adverse stimulus. The higher concentrations of NPs had a direct toxic effect on the L02 cells, leading to decreased viability. In general, NP exposure did not induce significant changes in viability in BEAS-2B cells, which is consistent with observations made in previous studies,^17,37^ except for a significant decrease at the highest concentration, indicating that NPs did not induce a large amount of cell death in BEAS-2B cells.

Internalization assays with and without fluorescent labeling were conducted to investigate whether NPs entered the cells. The fluorescent NPs were found to accumulate in the cytoplasm of the L02 and BEAS-2B cells in a dose-dependent manner. NP uptake was evident in the higher-NP-concentration groups (0.0625, 0.125, and 0.25 mg/mL). These results were similar to the internalization results obtained in previous studies in which fluorescent microplastics/NPs were incubated with other cell lines.^17,32,38^ The pigment from the fluorescent NPs may induce toxicity; therefore, to avoid any toxicity from the pigment leachate, the cells were incubated with nonfluorescent NPs in subsequent experiments, and the uptake of nonfluorescent NPs at selected concentrations was confirmed by TEM analysis (Figure 1B,D). The lowest concentration with an observed effect (0.0125 mg/mL) is lower than the reported NP concentrations of 0.04 mg/mL in the aquatic environment,^8^ suggesting the possibility of environmental significance. The estimated concentrations in units of particles/mL and the concentrations of internalized NPs (in units of particles/cell) are listed in Table 1; the associated calculation is provided in the Supporting Information. According to that calculation process, the previously reported NP concentration of 0.04 mg/mL in the aquatic environment is equal to ∼1.4 × 10^11^ particles/mL. Considering that TEM observations provide information on only a small area of a cell, the actual concentrations of internalized NPs may be higher than the estimation.

**Table 1:** Estimated Concentrations of NPs in Cells during the Exposure Period in Units of Particles/mL and Particles/Cell

nominal exposure concentrations (mg/mL)	estimated nominal exposure concentrations (particles/mL)	estimated concentrations after NP entering cells (particles/cell)
Human Liver L02 Cells		
control	ND^a^	ND^a^
0.0125	4.4 × 10^10^	1–2
0.125	4.4 × 10^11^	3–5

Human Lung BEAS-2B Cells		
control	ND^a^	ND^a^
0.0125	4.4 × 10^10^	1–3
0.25	8.9 × 10^11^	3–5

a ND: not detected.

The above results suggested that NP entered the cells. However, no lethal effects were observed, indicating that the effects in organelles, such as the perturbation of intracellular physiological processes, were sublethal. Previous studies have suggested that mitochondria may be sensitive to damage from NPs,^39,40^ as mitochondria are crucial organelles for metabolism and are required to maintain normal cell function.^41^ However, the toxicity mechanisms of NPs remain unclear. Therefore, we further explored whether NP exposure damaged mitochondria and investigated the underlying mechanisms by which NPs may cause damage.

### Mitochondrial Damage Induced by NPs

**Figure 2:**
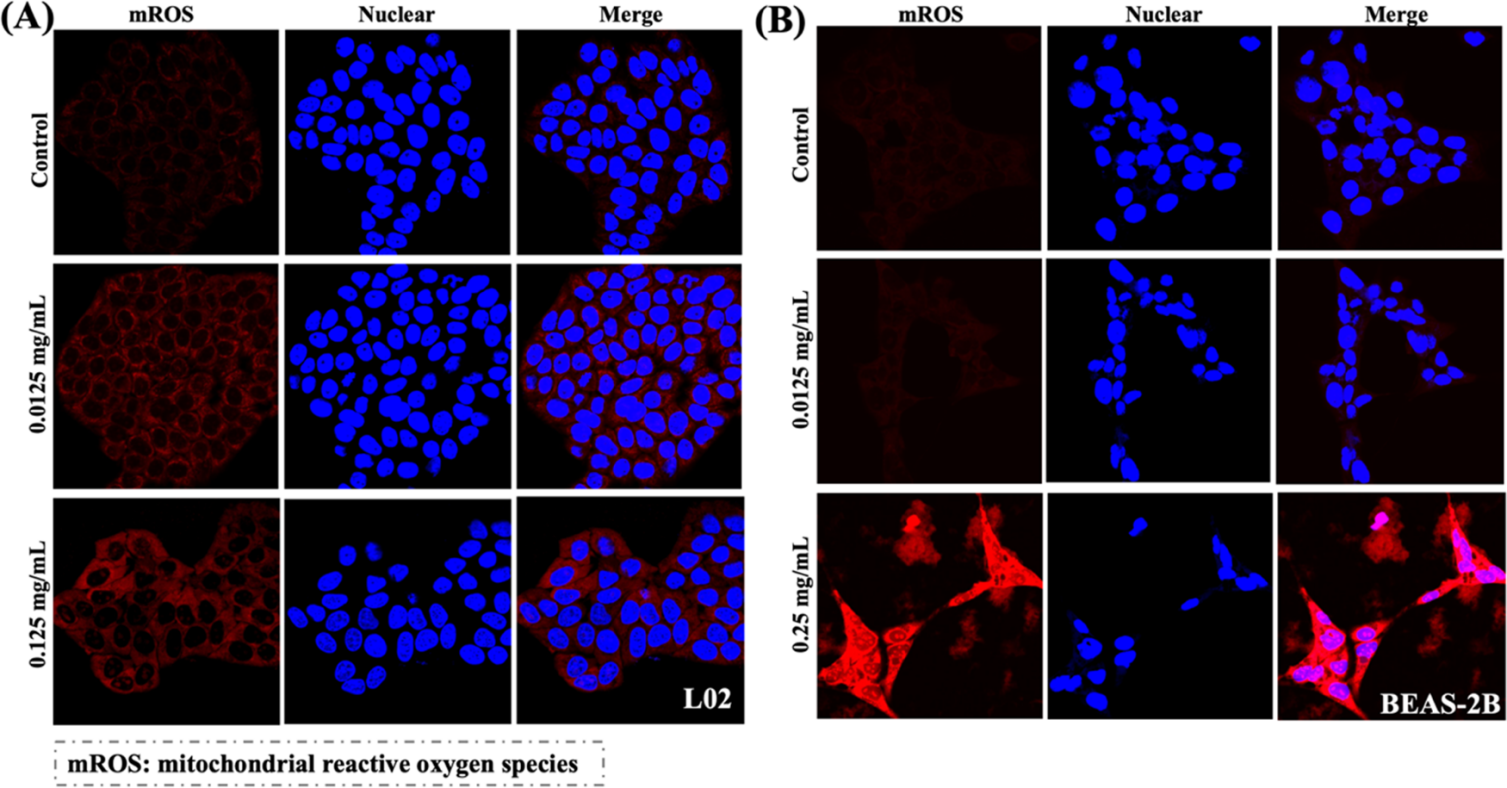
NP-induced mROS production in the normal human hepatic L02 cell line (A) and lung BEAS-2B cell line (B) (in 5 μM MitoSOX Red); *n* = 3.

The mROS production, MMP, and mitochondrial respiration were explored in L02 and BEAS-2B cells to examine whether NP exposure altered normal mitochondrial function. The overproduction of mROS has been recognized as one of the drivers of mitochondrial damage.^42,43^ In L02 cells, mROS production increased with NP treatment in a dose-dependent manner, with changes visible even after treatment with low concentrations of NPs (Figure 2A). These results were consistent with those reported in other cell lines.^17,44^ A slight increase in mROS production was observed in BEAS-2B cells after treatment with the same low concentration of NPs as that used to treat L02 cells (Figure 2B), but this change was less marked than that seen after this treatment in L02 cells. These differences in mROS production in hepatic and lung cells in response to NP treatment may be because the lung supplies the blood with high O_2_ concentrations, and this biological function might cause a greater antioxidant response in lung cells than in other cell lines.^45^ The results suggest that NP exposure contributed to the generation of a prooxidative environment, especially in the L02 cells.

**Figure 3:**
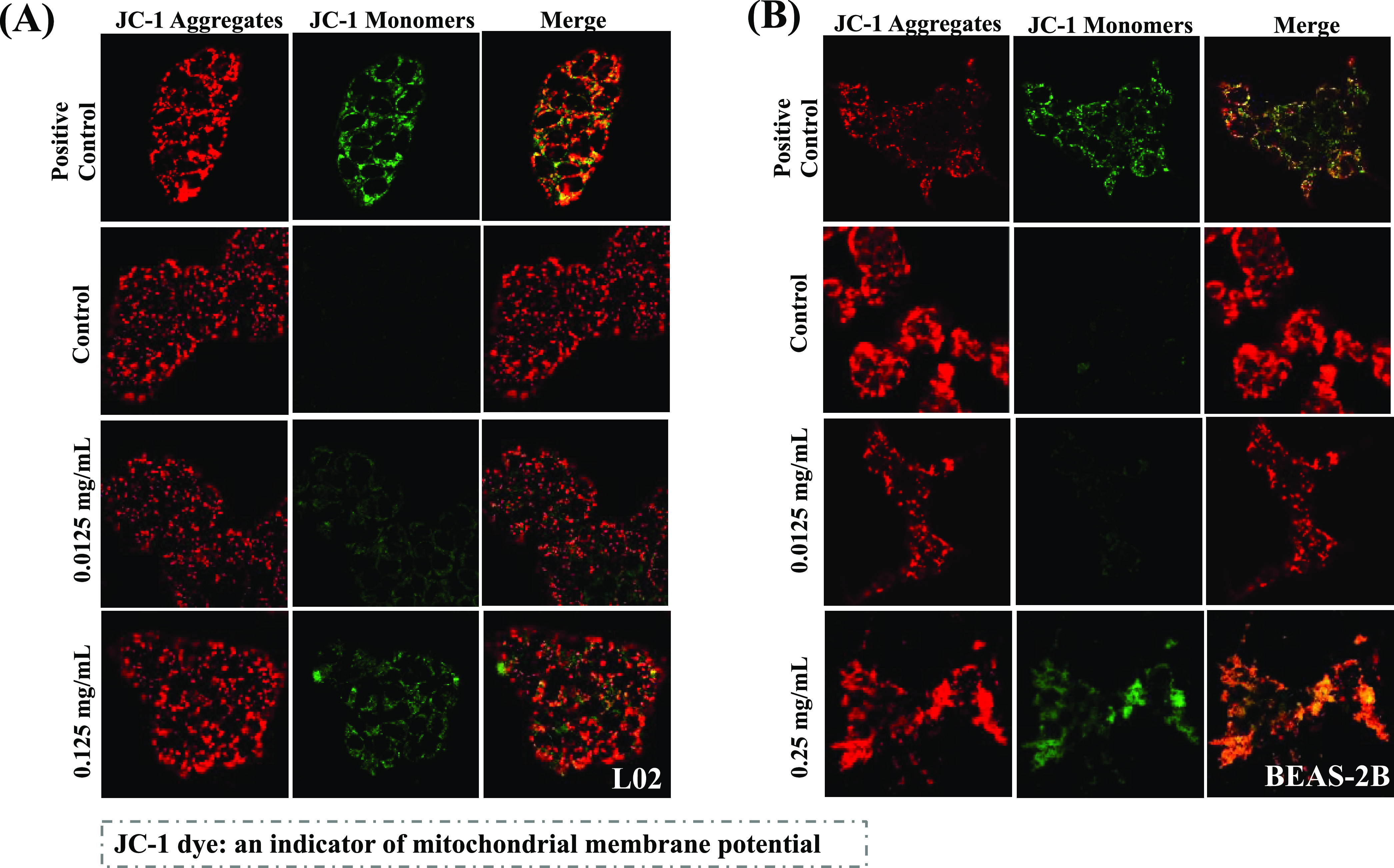
NP-induced MMP alterations in the normal human hepatic L02 cell line (A) and lung BEAS-2B cell line (B); *n* = 3.

The release of extra mROS in cells perturbs the mitochondrial membrane, thereby affecting the MMP and increasing mitochondrial damage.^46^ Thus, the MMP was examined using the cyanine dye JC-1 in L02 and BEAS-2B cells (Figure 3). When cells are healthy, JC-1 aggregates are formed and generate red fluorescence; in contrast, when cells are unhealthy, JC-1 remains in a monomeric form and generates green fluorescence. Thus, a transition from red to green fluorescence indicates an alteration in the MMP, which is an early indication of cell death and thus a marker of the functional status of the mitochondria. After exposure to low NP concentrations, a small number of JC-1 monomers were present in L02 cells, and the change was negligible in BEAS-2B cells. After exposure to high NP concentrations, JC-1 monomers were present in both cell lines, indicating the loss of membrane potential, particularly in the L02 cells. Mitochondrial homeostasis was also perturbed and mitochondrial respiration via the ETC could be further inhibited, the effects which have also been effectively demonstrated in animal studies.^39,47^

**Figure 4:**
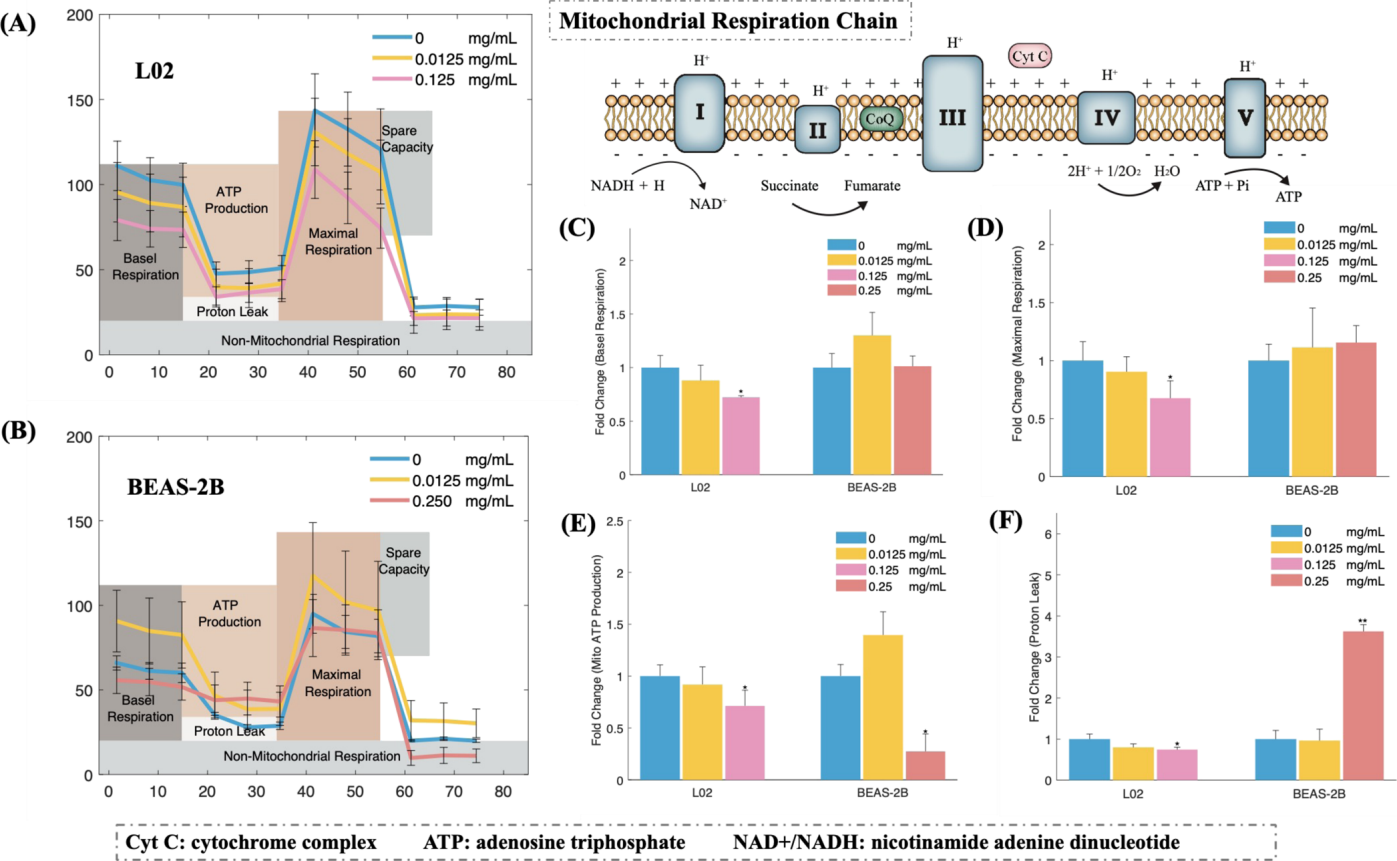
NP-induced mitochondrial stress responses in the normal human hepatic L02 cell line (A) and lung BEAS-2B cell line (B) through the mitochondrial respiration chain. Relative changes in key parameters of mitochondrial function were measured: basal respiration (C), maximal respiration (D), mitochondrial ATP production (E), and proton (H+) leakage (F); *n* = 3; *p < 0.05, **p < 0.01.

The changes in mitochondrial respiration after NP exposure, in terms of the effects on oxidative phosphorylation in the ETC, were further evaluated. Figure 4A,B shows the mitochondrial stress profiles in L02 and BEAS-2B cells after NP exposure. All measured parameters in the L02 cells followed a dose-dependent trend, with significant decreases in basal and maximal respiration (Figure 4C–F). These metrics were also decreased in BEAS-2B cells in the high-NP-concentration group; however, these changes were not significant (Figure 4C,D). ATP is the primary energy carrier in mitochondria,^48^ and considerable decreases in mitochondrial ATP production were observed in the L02 cells (in both NP-concentration groups) and BEAS-2B cells (in only the high-NP-concentration group) (Figure 4E). These decreases in ATP production would decrease cell energy levels, thereby possibly inhibiting respiration. However, in BEAS-2B cells exposed to low NP concentrations, mitochondrial ATP production increased, possibly because of a stress reaction to the NPs.

These results show that the NPs had a greater inhibitory effect on mitochondrial respiration in hepatic cells than in lung cells. This difference may be due to the different characteristics of hepatic and lung cells and their different stress-reaction intensities. The inhibitory effect on mitochondrial respiration might trigger a chain of subsequent reactions, resulting in the disruption of ATP synthesis in the mitochondrial inner membrane. The inhibition of the ETC was also a major reason for the altered mROS production and MMP in both cell lines, as electron flux through the ETC controls these functionalities. Associations between NP exposure and ETC interference have been reported, such as a decrease in the mitochondrial coupling efficiency and the disruption of mitochondrial energy generation in cells from humans^17,49^ and other species,^39,50^ but the underlying mechanisms of these NP-mediated effects remain unclear. Therefore, considering that mitochondrial respiration occurs via complexes I–V in the ETC, which uses different metabolites,^51^ we used nontargeted metabolomics to profile potential metabolic pathways and thus identify the toxicity mechanisms at the molecular level. The nontarget analysis is also a useful approach for clarifying whether mitochondria are the most vulnerable cellular target of NP exposure.

### Metabolic Profiling, Endogenous Biomarkers, and Related Disturbed Metabolic Pathways

A global MS-based nontarget metabolomics approach enabled the measurement of endogenous metabolites and the identification of specific toxicity pathways in response to NP exposure. The PLS-DA models showed satisfactory fitness and predictive power of extracted MS features for L02 cells (Figure S2A) and BEAS-2B cells (Figure S2B) in both positive and negative modes (R2Y and Q2 > 0.5). Significant differences were observed in the NP-treated groups and depended on the NP concentration, implying that NP exposure induced perturbations in the metabolic profiles of the two cell lines in a dose-dependent manner. This finding is in accordance with the results shown in the volcano plots (Figure S3). The metabolic alterations were more severe in the L02 cells than in the BEAS-2B cells, as indicated by the greater number of feature changes in the L02 cells at a low NP concentration (0.0125 mg/mL) (Figure S3A,C). These results imply that the metabolic functions of hepatic cells are more vulnerable than those of lung cells to low concentrations of NPs.

**Figure 5:**
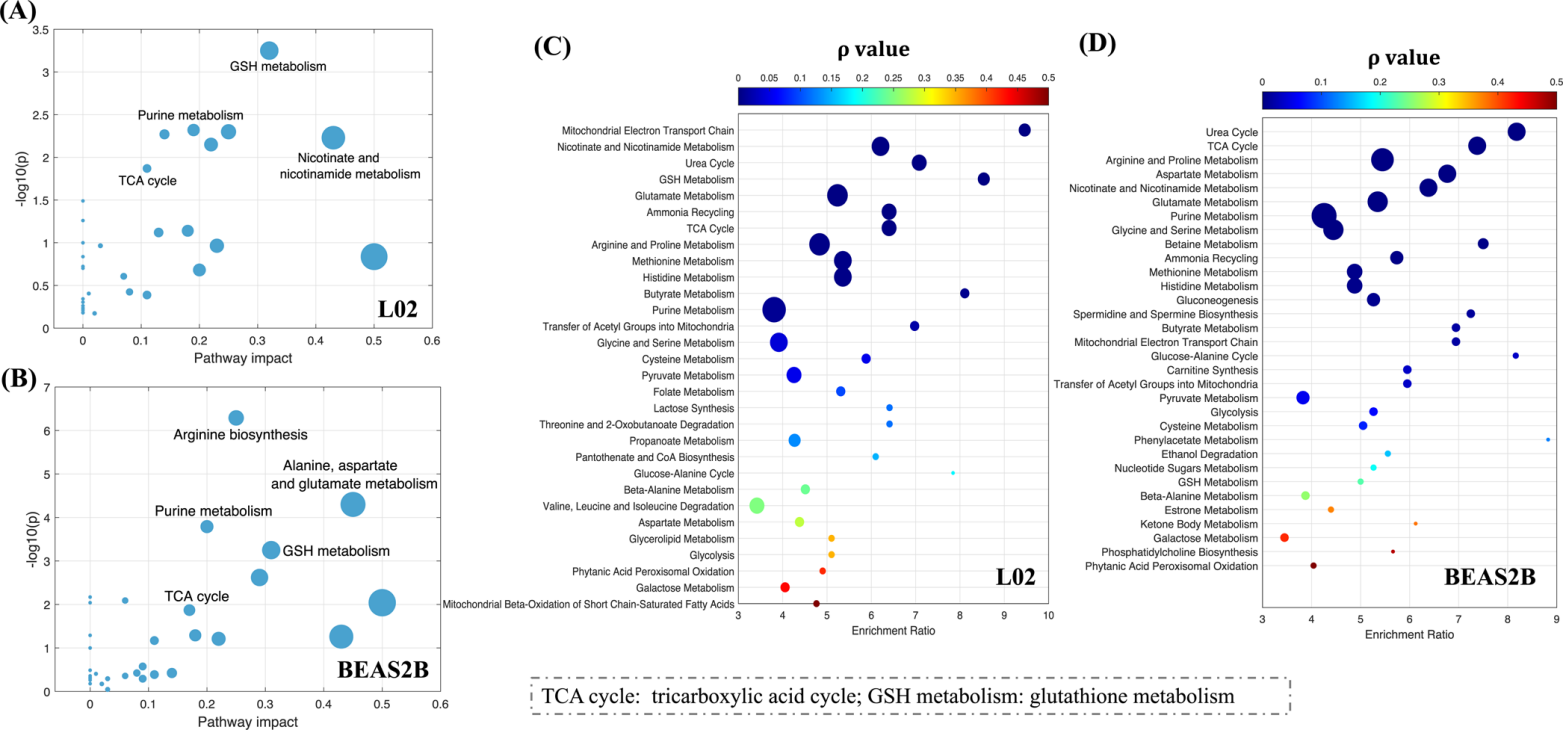
Metabolic pathway analysis and enrichment analysis of the most relevant metabolite sets in the NP-treated normal human hepatic L02 cell line (A, C) and lung BEAS-2B cell line (B, D).

More than 35 endogenous biomarkers were identified in each cell line by searching mass spectral libraries; these results are presented in Table S2 (L02 cells) and Table S3 (BEAS-2B cells). These compounds were mainly nucleotides, nucleosides, amino acids, peptides, and carboxylic acids. The results of metabolic pathways showed that NP exposure affected nicotinate and nicotinamide metabolism in L02 cells and arginine biosynthesis and alanine, aspartate, and glutamate metabolism in BEAS-2B cells (Figure 5A,B). Enrichment analysis of the functional and biological patterns showed that the biological processes of the urea cycle and ETC that occur in the mitochondria were highly perturbed in both cell lines (Figure 5C,D). Additionally, in both cell lines, the three pathways most impacted by NP exposure were identified as mitochondrial-related pathways. These pathways were related to the tricarboxylic acid (TCA) cycle, glutathione (GSH) metabolism, and purine metabolism (Figure 5A,B), demonstrating that NP exposure impacted mitochondrial function.

### Disruption of Mitochondrial-Related Toxicity Pathways

**Figure 6:**
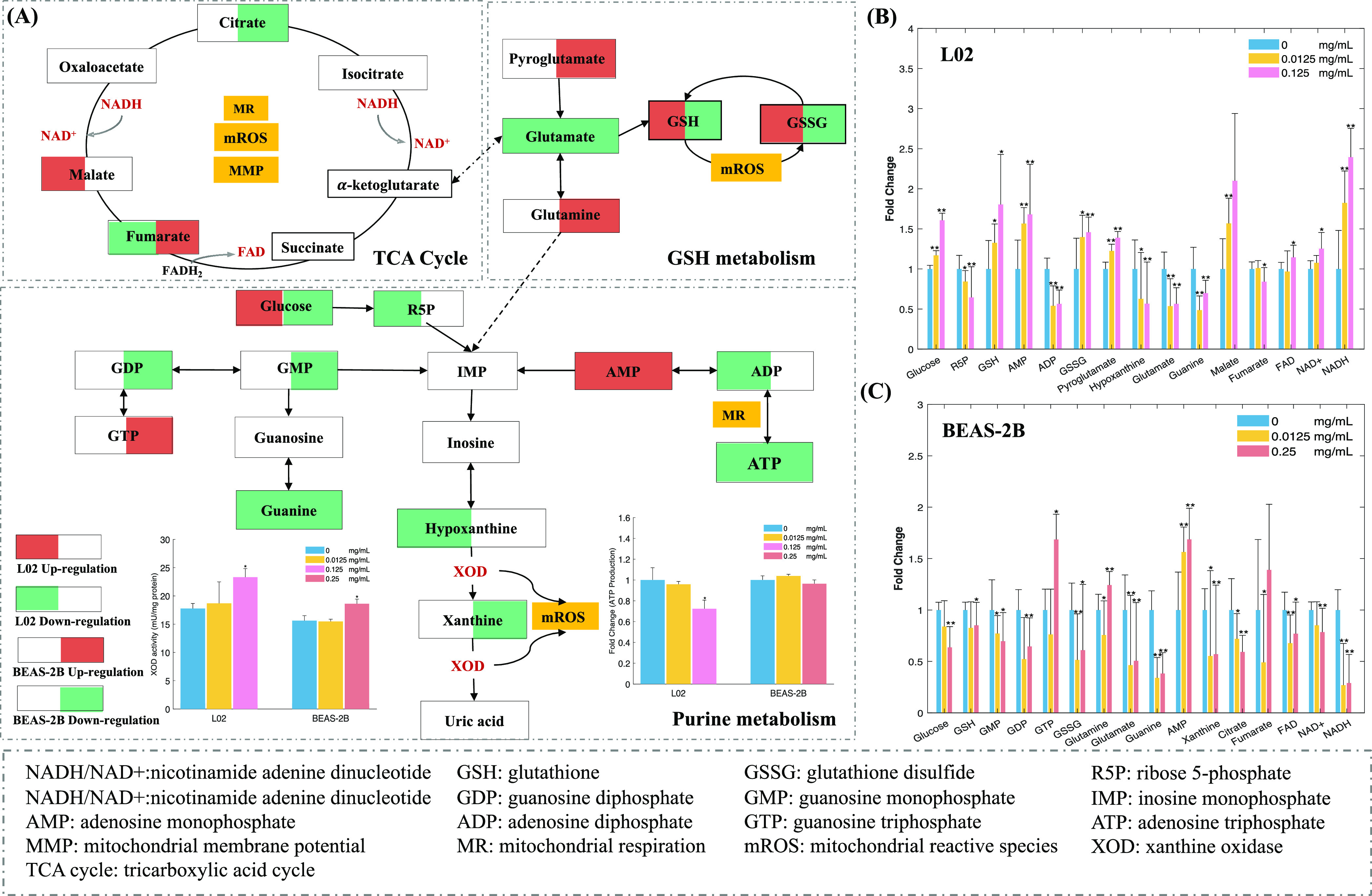
NP perturbed metabolic pathways of the TCA cycle, GSH metabolism, and purine metabolism (A). Relative changes in identified endogenous biomarkers in the normal human hepatic L02 cell line (B) and lung BEAS-2B cell line-2B (C), *n* = 9; for XOD activity, *n* = 6; *p < 0.05, **p < 0.01.

The TCA cycle is central to cellular energy metabolism and supplies energy for cellular respiration.^52,53^ NP-treated L02 cells showed increases in the contents of some endogenous biomarkers of the TCA cycle, such as malate, and slight decreases in the contents of others, such as fumarate (Figure 6). After treatment with high concentrations of NPs, BEAS-2B cells exhibited markedly decreased contents of citrate but increased contents of fumarate (Figure 6B,C). These results are consistent with a previous study that found that NP-treated BEAS-2B cells exhibited increased levels of TCA intermediate metabolites.^24^

Furthermore, the accumulation of fumarate can contribute to increases in mROS concentrations, which causes an imbalance in the mitochondrial respiratory chain.^54,55^ The intracellular coenzymes nicotinamide adenine dinucleotide NAD+ and NADH play a vital role in the final step of ATP production in the ETC because they are key electron donors and acceptors that transfer electrons during mitochondrial respiration.^56^ An imbalance between NAD+ and NADH in the TCA cycle, as observed in both cell lines (Figure 6), would perturb ATP generation and inhibit respiration, both of which are hallmarks of mitochondrial dysfunction.^56^

Purine metabolism is one of the main metabolic processes of cellular ATP production, and alterations of ATP can lead to changes in the metabolic phenotype.^57^ We observed that ATP concentrations were significantly decreased in L02 cells after NP treatment in a dose-dependent manner, which is in line with the observed changes in ATP generation by the mitochondrial ETC. A similar decrease in ATP concentrations was also observed in BEAS-2B cells treated with a high concentration of NPs, but these changes were not significant. The significant reduction in ATP levels in L02 cells may account for their higher sensitivity than BEAS-2B cells to NP-induced mitochondrial damage. Moreover, insufficient adenosine diphosphate (ADP, the less energy-rich precursor to ATP) production would inhibit ATP generation (Figure 6A), resulting in a series of ETC abnormalities, such as alterations in the MMP.

In the high-NP-concentration group of both cell lines, there were decreases in the contents of most of the other downstream endogenous biomarkers of the purine metabolism pathway (Figure 6). These results suggest that the purine pathway was significantly downregulated by this NP treatment. Two essential metabolites, hypoxanthine and xanthine, are substrates of XOD, which catalyzes the formation of uric acid and generates hydrogen peroxide and superoxide anions (oxidized products).^58^ XOD activity was significantly increased after NP exposure in both cell lines but more so in the L02 cells (Figure 6A), indicating that XOD is critical for mROS production.

A disturbance in GSH metabolism was also responsible for the NP-induced imbalance in the mitochondrial antioxidant system. The intracellular redox potential is strongly determined by GSH.^59^ Approximately 15% of cellular GSH is located in the mitochondria, where it plays an essential role in protecting mitochondria from any excess mROS produced by the ETC.^60^ The significant upregulation of GSH in L02 cells by NP treatment, which occurred in a dose-dependent manner, can be considered an adaptive response because it would have enhanced the antioxidant defense system (Figure 6A,B). The dramatic decrease in GSH and glutathione disulfide (GSSG) contents in BEAS-2B cells exposed to high NP concentrations clearly indicated that this treatment altered the cellular oxidation state (Figure 6A,B). These differences in GSH contents in the two cell lines highlight their distinct mROS responses to NP exposure. In addition, the excess pyroglutamate contents observed in BEAS-2B cells were associated with GSH depletion,^61^ which could be an indication of higher oxidative stress in BEAS-2B cells treated with a high concentration of NPs. These alterations in the concentrations of the endogenous metabolites of GSH metabolism demonstrated the redox status of cells due to NP treatment; specifically, a prooxidant condition was generated in the mitochondria in response to NP stimulation.

This study, for the first time, provides new insights for understanding the mitochondrial-related response pathways and metabolic mechanisms in normal human cells in response to NP exposure. In particular, the results show that NPs can enter cells and induce mitochondrial damage without causing mass cell death. Because mitochondrial functions are closely connected to metabolic changes, nontarget metabolomics was used to confirm that mitochondria are the most sensitive organelles to NP exposure in both L02 cells and BEAS-2B cells and also revealed their metabolic mechanisms of NP toxicity. The metabolic functions of L02 cells were more vulnerable than BEAS-2B cells to a low NP concentration that might be comparable to the reported environmental NP concentration. The effects observed at the molecular and cellular levels could be considered predisease events to predict NP exposure outcomes at the tissue and organ levels. Thus, the mitochondrial damage observed in this study might cause cells and eventually organ tissues to malfunction. Moreover, pure commercial NPs without additional chemicals were used in this study, and these NPs showed potential risks to both cell lines, which demonstrates the previously underappreciated possible health impacts of NPs on different organs. Considering that NP pollution is complex, owing to variations in NP shape, age, and length of coexistence with other toxic chemicals in the environment, the actual adverse impacts of NPs on environmental and human health are likely to be greater than those measured in this study. Therefore, a better understanding of the potential adverse effects of NP exposure in humans is needed.

## Supporting Information Available

Experimental details of 80 nm NP characterization by TEM, cellular TEM analysis, mitochondrial stress test, and estimation process of NP number concentrations; figures and tables address the following: (1) characterization of 80 nm NPs, (2) PLS-DA score plots of nontarget metabolomics data, (3) volcano plots of nontarget metabolomics data in L02 and BEAS-2B cells, (4) instrumental method for the analysis of metabolomics samples by UHPLC-QE Orbitrap MS, and (5) identification of significantly changed metabolites in hepatic L02 and lung BEAS-2B cells (PDF)
