# Supplementary material for: Metabolomics Reveal Nanoplastic-Induced Mitochondrial Damage in Human Liver and Lung Cells
Source: Environ Sci Technol. 2022 Aug 25;56(17):12483–93. doi: 10.1021/acs.est.2c03980 (PMC9454251; doi:10.1021/acs.est.2c03980)
Supplement: Supplementary file 1 — es2c03980_si_001.pdf [file es2c03980_si_001.pdf]

Supplementary Information for:

Metabolomics Reveal Nanoplastic-Induced Mitochondrial Damage in Human Liver and Lung Cells

Siyi Lin[a,b], Hongna Zhang[a], Chen Wang[c], Xiu-Li Su[a], Yuanyuan Song[a], Pengfei Wu[a], Zhu Yang[a], Ming-Hung Wong[d], Zongwei Cai[a,*], Chunmiao Zheng[b,*]

[a]*State Key Laboratory of Environmental and Biological Analysis, Department of Chemistry, Hong Kong Baptist University, Hong Kong SAR, P. R. China*

[b]*State Environmental Protection Key Laboratory of Integrated Surface Water-Groundwater Pollution Control, School of Environmental Science and Engineering, Southern University of Science and Technology, Shenzhen 518055, P. R. China*

[c]*State Key Laboratory of Environmental Criteria and Risk Assessment, Chinese Research Academy of Environmental Sciences, Beijing 100012, China*

[d]*Consortium on Health, Environment, Education and Research (CHEER), Department of Science and Environmental Studies, The Education University of Hong Kong, Hong Kong, China*

[*]Corresponding authors.

Email Address:

zwcai@hkbu.edu.hk (Prof. Zongwei Cai) Tel: +852-34117070;

zhengcm@sustech.edu.cn (Prof. Chunmiao Zheng) Tel: +86-0755-88018086

Total pages: 14

Numbers of Figures: 3

Numbers of Tables: 3

Characterization of 80nm-NP. The commercial NP standard was diluted with 50% ethanol solution and sonicated for 10 min. An approximate 10 μL of the suspension was dropped on the nickel mesh with ultra-thin carbon film and dried in a 60°C-drying oven for 15 min. Then the samples were observed via transmission electron microscopy (TEM, TEM, FEI Talos F200X, Thermo Fisher).

Cellular TEM Analysis. The resulting fixed cells were washed with PBS (0.1 M), osmicated with 1% osmium tetroxide for 1–2 h, and then washed again with PBS. The samples were dehydrated with ethanol solutions with a gradient of concentrations for 15 min, transferred to a mixed solution of embedding medium and acetone (3:1 v/v) and incubated for 3 h. Cell sections with a thickness of 70–90 nm were obtained using a Leica EM UC7 ultramicrotome and used for TEM observation (JEM1200EX, Jeol).

Mitochondrial Stress Test. Prior to this test, the sensor cartridges were hydrated (200 μL/well) in a non-CO_2 incubator (37°C) overnight. The Agilent Seahorse Mito Stress Test Kit components included oligomycin and carbonyl cyanide 4-(trifluoromethoxy)phenylhydrazone (FCCP) and rotenone and antimycin A (ROT/AA). A mix of sufficient concentrations of oligomycin (L02: 1.5; BEAS-2B: 1 μM), FCCP (L02: 2; BEAS-2B: 1 μM), and ROT/AA (L02 and BEAS-2B: 1 μM) were loaded into the injection ports of the hydrated sensor cartridge to give the optimized cartridge concentrations. The hydrated sensor cartridge was then transferred to a Seahorse XFp Analyzer (Agilent Technologies) to initiate calibration (~20 min). After calibration, the utility plate was removed and replaced with the cell miniplate, and the assay was performed. The first injection comprised oligomycin, which decreases electron flow through the electron transport chain (ETC), reducing the oxygen consumption rate and inhibiting the final complex (V) of adenosine triphosphate (ATP) synthase. The second injection comprised the coupling agent (FCCP), which collapses the proton gradient and disrupts the MMP, allowing the mitochondrial maximal capacity to be determined. The final

injection comprised rotenone, which inhibits complex I, and antimycin A, which inhibits complex III, thereby completely inhibiting mitochondrial oxygen consumption and the ETC.[1] Five indicators were then measured: (1) ATP production; (2) basal respiration (energy demand under baseline conditions); (3) maximal respiration (i.e., maximum rate of cellular respiration); (4) spare respiratory capacity (i.e., the cells' capability to respond to increasing energy demand); and (5) proton leakage, which indicates remaining basal respiration (i.e., that not coupled to ATP production). The results were normalized by cell numbers per well.

Estimation of NP number concentrations. The inconsistent methods of characterization and quantification of NP in the environmental samples, food items or other subjects lead to various units of abundance, mainly items or particles per unit sampling area, weight or volume,[38,39] making it hard to compare to exposure concentration. We, therefore, estimated the concentrations of 80-nm spheroidal NPs through the following calculations, based on the well-known Archimedes' principle.

$$\mathrm{r}=40\ \mathrm{nm};\ \rho=1.05\ \mathrm{g/cm^3}$$

$$V=\frac{4}{3}\pi r^3$$

$$m=\rho V=\frac{4}{3}\pi\rho \mathrm{r}^3 = 2.8\times10^{-16}\ \mathrm{mg}$$

where r is the radius of NP, 40 nm; ρ is the water density, 105 g/cm^3; V is the volume; and m is the mass, 2.8×10^{-16}. Therefore, the NP stock solution approximately contain 8.9 x10^{10} items/L (25 mg/mL). The low concentrations (0.0125 mg/mL) used in both human hepatic L02 and lung epithelial BEAS-2B cells approximately are 4.4 $\times10^{10}$ particles/mL, the high concentration (0.125 mg/mL) used in L02 approximately are 4.4×10^{11} particles/mL and the high concentration (0.25 mg/mL) used in BEAS-2B approximately are 8.9×10^{11}particles/mL.

1. Supplemental Figures

Figure Captions

**Figure S1**	Characterization of 80nm-NP by TEM
**Figure S2**	PLS-DA score plots of metabolomics data in hepatic L02 cells and lung BEAS-2B cells
**Figure S3**	Volcano plots of metabolomics data in hepatic and lung cells

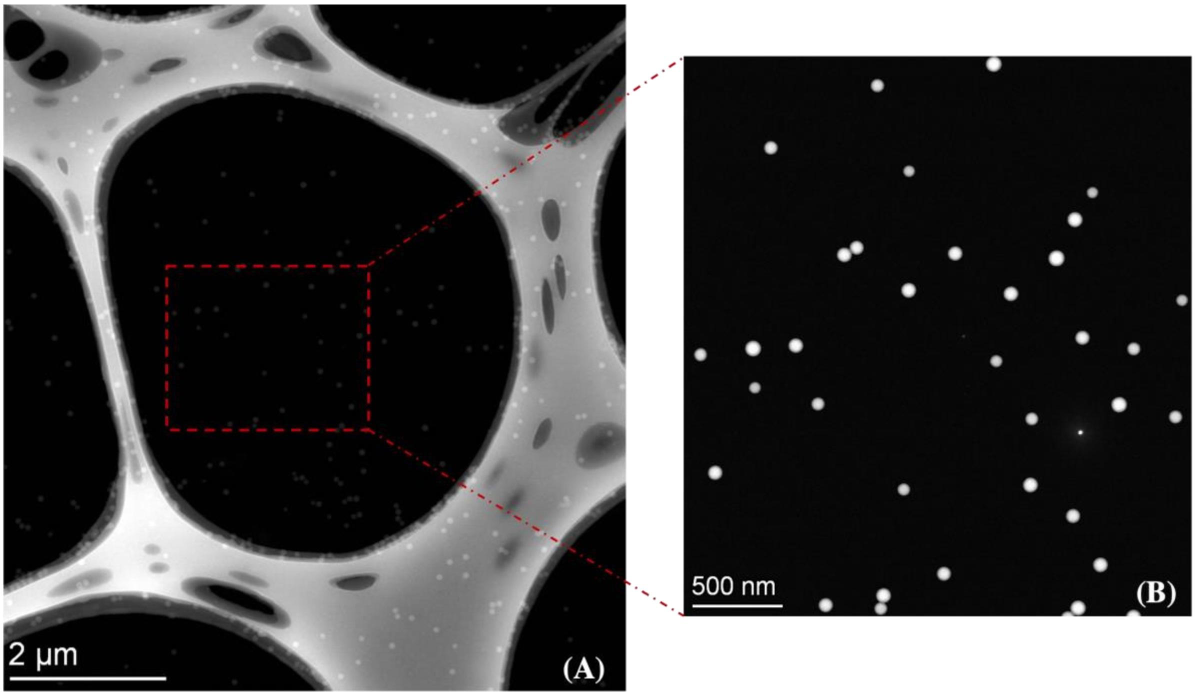

Figure S1. Characterization of 80nm-NP by TEM at different scale bars: (A) 2 μm and (B) 500 nm.

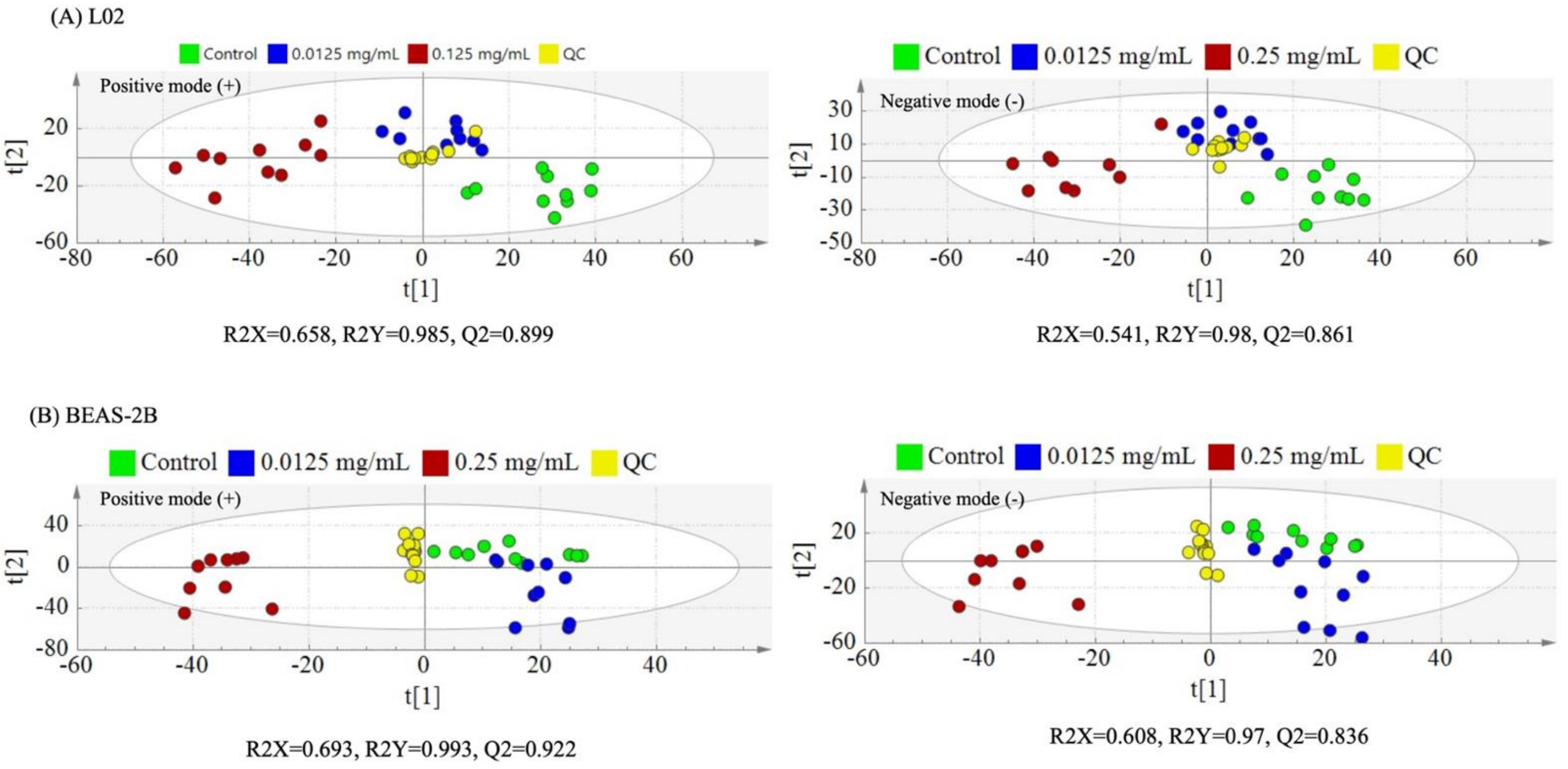

Figure S2. PLS-DA score plots of metabolomics data in hepatic L02 cells (A) and lung BEAS-2B cells (B). n=9.

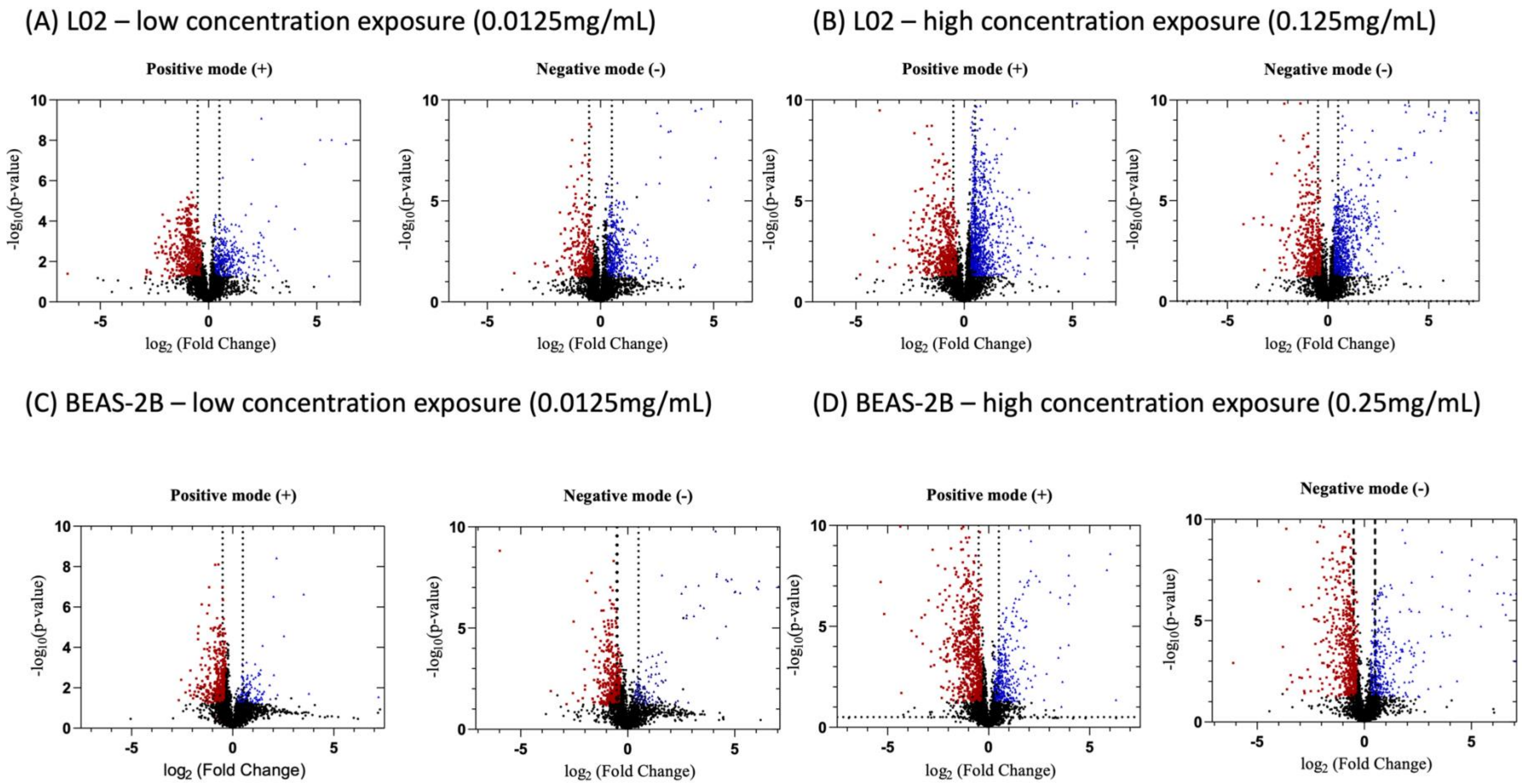

Figure S3. Volcano plots of metabolomics data in hepatic L02 cells (A, B) and lung BEAS-2B cells (C, D). Blue colour represented metabolic features with FC<0.8, the red colour represented metabolic features with FC >1.2, both *p* values <0.05, n=9.

2. Supplemental Tables

Table Captions

**Table S1**	Instrumental method for the analysis of nontarget metabolomics samples by UHPLC-QE Orbitrap MS
**Table S2**	Identification of significantly changed metabolites in hepatic L02 cells
**Table S3**	Identification of significantly changed metabolites in lung BEAS-2B cells

Table S1. Instrumental method for the analysis of nontarget metabolomics samples by UHPLC-QE Orbitrap MS

Instrument	Thermo Scientific Ultrahigh-performance liquid chromatography (UHPLC)-Q Exactive Focus Hybrid Quadrupole-Orbitrap Mass Spectrometer (QE Orbitrap MS) system
Analytical Column	ACQUITY UPLC BEH amide column (100 × 2.1 mm; 1.7 μm)
Column Temperature	40 °C
Mobile Phases	Positive mode:<br>A. 100% water + 10 mM ammonium formate and 0.125% formic acid<br>B. 95% acetonitrile +5% water + + 10 mM ammonium formate and 0.125% formic acid<br>Negative mode:<br>A. 100% water + 10 mM ammonium formate+0.04% ammonium hydroxide<br>B. 95% acetonitrile +5% water+10 mM ammonium formate+0.04% ammonium hydroxide
Flow rate	300 μL/min
Gradient Profile	0–2 min: 100% B;<br>2–7.7 min: 100% B–70% B;<br>7.7–9.5 min: 70% B–40% B;<br>9.5–10.3 min: 40% B–30% B;<br>10.3–12.3 min: 30% B;<br>12.3–14.8 min: 30% B–100% B;<br>14.8–20 min: 100% B
Injection Volume	10 μL
MS Parameters	Capillary voltage: 3.6 kV (positive); −2.6 kV (negative) ion mode;<br>Capillary temperature: 350 °C;<br>Probe heater temperature: 320 °C; Sheath gas flow: 40 arbitrary units;<br>Aux gas flow: 10 arbitrary units;<br>Sweep gas flow: 1 arbitrary unit;<br>High-resolution full scan mode (mass resolution: 35000)
MS/MS parameters	Mass resolution: 17500; Dynamic exclusion duration: 3.5 s;<br>Normalized collision energy: 30 ± 5;<br>Activation Q: 0.25; AGC target: 1×10^5;<br>Maximum injection time: 100 ms;<br>Loop counts, 3; Isolation window, 1.8 *m/z*.

Table S2 Identification of significantly changed metabolites in hepatic L02 cells

No.	Compounds	RT/min	m/z	MS pattern	FC (H/C)	*p* value	Related pathways
1	ADP	10.73	428.0971 (+)	97.02841, 136.06174, 137.06648, 428.03656	0.33	3.48E-03	Purine metabolism
		10.45	426.0836 (-)	78.95751, 134.04591, 158.92410, 328.04761	0.56	8.66E-08	
2	ACh	8.47	146.1021 (+)	60.08099, 87.04395, 146.11719	0.34	7.17E-03	Glycerophospholipid metabolism
		10.09	306.0766 (-)	128.03448, 143.04477, 254.07870, 272.08936	1.80	5.75E-02	
3	MTA	10.11	298.1002 (+)	75.02630, 97.02833, 136.06172, 163.04265	0.47	1.85E-04	Cysteine and methionine metabolism
4	Niacin	8.48	124.0391 (+)	53.03888, 68.04964, 80.04935, 96.04424	0.47	4.28E-03	Nicotinate and nicotinamide metabolism
5	Nicotinamide	1.52	123.0550 (+)	53.03886, 80.04932, 96.04420, 79.04141	0.52	2.25E-04	Nicotinate and nicotinamide metabolism
6	deamino-NAD+	10.71	665.0988 (+)	97.02837, 136.06174, 232.08264, 428.03619	0.53	6.13E-07	Nicotinate and nicotinamide metabolism
7	Glutamate	8.48	148.0196 (+)	56.04976, 84.04765, 85.02804, 102.05503	0.56	5.27E-05	Glutathione metabolism; Histidine metabolism
8	Hypoxanthine	4.35	137.0455 (+)	55.02932, 82.03976, 94.06496, 110.03461,	0.57	1.33E-02	Purine metabolism
9	Hydroxycinnamates	8.14	165.0131 (+)	65.03873, 77.03847, 91.05421, 95.04908	0.58	5.61E-03	Phenylalaninen metabolism
10	Pantothenic Acid	8.39	218.1032 (-)	71.04871, 88.03892, 146.08102,147.08452	0.62	3.22E-03	Pantothenate and CoA biosynthesis
11	Cystathionine	8.94	223.0118 (+)	56.04974, 88.02139, 135.03040, 134.02682	0.63	3.53E-06	Cysteine and methionine metabolism
12	R5P	9.97	229.0199 (-)	78.95741, 96.96818, 138.97888, 211.00046	0.64	2.65E-03	Purine metabolism
13	Guanine	6.46	152.0564 (+)	55.02935, 82.03973, 107.03512,110.03475	0.69	7.07E-03	Purine metabolism
14	Fumarate*	9.69	115.0022 (-)	71.0132	0.84	2.16E-02	Oxidative phosphorylation; TCA cycle
15	FAD	9.54	784.1512 (-)	96.9680, 180.9899, 346.0565	1.14	3.24E-02	Riboflavin metabolism; TCA cycle
16	UDP-GlcNAc	10.50	608.0874 (+)	138.05476, 204.08627, 186.07597, 405.00677	1.22	2.60E-04	Amino sugar and nucleotide sugar metabolism
		9.92	606.0744 (-)	96.96823, 128.92393, 176.93521, 272.95718	1.26	7.41E-04	
17	NAD+	10.10	662.1026 (-)	137.06595, 136.06171, 232.08261, 348.06955	1.25	1.60E-02	Oxidative phosphorylation, TCA cycle
18	CMP	10.11	324.0583 (+)	69.04468, 95.02376, 97.02835, 112.05034	1.28	1.59E-02	Nucleotide metabolism
19	GDP-mannose	10.32	604.0702 (-)	78.95753, 362.05237, 424.00882, 443.01935	1.33	1.97E-04	Amino sugar and nucleotide sugar metabolism
20	Arginine	10.14	175.1186 (+)	60.055562, 70.06512, 116.07034, 130.09773	1.36	1.15E-07	Aminoacyl-tRNA biosynthesis
21	Cyclic ADP-ribose	10.01	540.9455 (-)	78.95748, 134.04581, 272.95718, 346.05606	1.38	5.16E-04	Calcium signalling pathway
22	Pyroglutamate	10.14	130.0973 (+)	56.04974, 84.04423,130.04973, 85.02834	1.39	2.87E-07	Glutathione metabolism
23	UMP	10.04	323.0285 (-)	78.95741,96.96810, 111.01838, 192.99045	1.39	3.09E-03	Nucleotide metabolism

24	NAAG	9.92	303.0353 (-)	96.00770, 128.03418, 146.04472, 182.04547	1.4	6.43E-05	Alanine, aspartate and glutamate metabolism
25	GlcN-6-P	10.31	258.1038 (-)	78.95741, 96.96809, 101.02249, 241.01228	1.4	2.35E-02	Alanine, aspartate and glutamate metabolism
26	GDP-fucose	10.07	588.0333 (-)	78.95749, 150.04128, 258.92389, 344.03967	1.44	8.75E-03	Fructose and mannose metabolism
27	GSSG	9.03	611.1259 (-)	128.03412, 143.04486, 254.07814, 306.07675	1.46	9.8E-03	Glutathione metabolism
28	Propionylcarnitine	6.95	218.1382 (+)	60.08096, 57.03373,85.02824, 144.10124	1.48	2.78E-04	Oxidation of branched chain fatty acids
29	UDP	10.27	403.0014 (-)	78.95746, 158.92403, 192.99907, 272.95734	1.48	2.41E-02	Pyrimidine metabolism
30	Cytosine	8.76	112.9996 (+)	52.01849, 68.01315, 67.02910, 95.02396	1.50	1.99E-02	Pyrimidine metabolism
31	Isoleucine	8.18	132.1011 (+)	57.05752, 69.06991, 86.09631	1.54	5.37E-05	Valine, leucine and isoleucine degradation
32	Glucose	9.63	179.0551 (-)	59.01288, 71.01300, 85.02806	1.61	1.38E-09	Glucose metabolism; glycolysis
33	AMP	9.51	348.0694 (+)	69.03345, 97.02827, 119.03509, 136.06160	1.68	6.03E-03	Purine metabolism
34		9.93	346.2205 (-)	78.95728, 96.96812, 134.04582, 192.98972	1.95	7.23E-04	
35	Homoserine	8.89	120.1030 (+)	56.04976, 74.05989,102.05477	1.93	5.32E-03	Biosynthesis of amino acids
36	Spermidine	11.24	146.1649 (+)	58.06536, 72.08080, 84.08070, 112.11193	2.01	4.80E-04	Glutathione metabolism
37	xylulose-5-P	9.26	229.0804 (-)	78.95741, 96.96818, 138.97888, 211.00046	2.09	2.11E-04	Hexose monophosphate pathway
38	Malate	10.09	133.0129 (-)	71.01302, 72.99236, 89.04292, 115.00207	2.10	8.5E-02	TCA cycle
39	MEH	9.55	170.0572 (+)	68.04964, 83.06035, 97.07613, 109.07612	2.14	5.74E-06	Histidine metabolism
40	NADH	9.58	664.1182 (-)	408.0118, 397.0210, 346.0558	2.39	4.98E-04	Oxidative phosphorylation; TCA cycle

* confirmed by commercial standards (confidence level: level 1).

Abbreviations: ADP, adenosine diphosphate; Ach, acetylcholine; MTA, methylthioadenosine; GSH, reduced glutathione; deamino-NAD+, nicotinic acid adenine dinucleotide; R5P, ribose 5-phosphate; FAD, flavin adenine dinucleotide; NAD+, nicotinamide adenine dinucleotide; UDP-GlcNAc, UDP-*N*-acetylglucosamine; CMP, cytidine monophosphate; UMP, uridine monophosphate; NAAG, N-Acetylaspartylglutamic acid; GlcN-6-P, glucosamine 6-phosphate GSSG, oxidized glutathione; UDP, uridine diphosphate; AMP, adenosine monophosphate; xylulose-5-P, xylulose-5-phosphate; MEH, methylhistidine; NADH, reduced nicotinamide adenine dinucleotide.

Table S3 Identification of significantly changed metabolites in BEAS-2B cells

No.	Compounds	RT/min	m/z	MS pattern	FC(H/C)	*p* value	pathway
1.	NADH	9.59	664.1183 (-)	408.0118, 397.0210, 346.0558	0.29	2.8841E-07	Oxidative phosphorylation
2	Guanine	7.39	152.0565 (+)	55.02939, 80.02434, 93.00839, 110.03501	0.38	8.9497E-08	Purine metabolism
3	UDP-galactose	10.85	566.6245 (-)	211.00243, 158.92375, 241.01292, 323.02869	0.48	1.6451E-08	Amino sugar and nucleotide sugar metabolism
4	ASA	9.24	291.1165 (+)	70.06518, 88.03922, 116.07053, 176.06667	0.50	1.0724E-08	Alanine, aspartate and glutamate metabolism
5	Glutamate	9.47	148.0130 (+)	56.04977, 85.02850, 84.04430, 102.05489	0.51	4.25E-03	Glutathione metabolism
6	Xanthine	7.39	153.0599 (+)	110.0349, 81.0084, 55.0294	0.57	9.15E-03	Purine metabolism
7	NAAG	9.94	303.0352 (-)	96.00761, 128.03401, 146.04457, 182.04503	0.59	3.9918E-07	Alanine, aspartate and glutamate metabolism
8	Citrate	9.07	191.0671 (-)	57.03358, 87.00718, 85.02778, 111.00712	0.59	5.74E-03	TCA cycle
9	GSSG	9.71	611.1089 (-)	128.03392, 143.04466, 254.07808, 272.08887	0.61	1.73E-02	Glutathione metabolism
10	UDP-GlcNAc	10.53	608.0880 (+)	138.05486, 204.08656, 186.07613, 405.00879	0.62	1.0725E-07	Amino sugar and nucleotide sugar metabolism
		10.33	605.0739 (-)	78.95748, 96.96800, 176.93486, 282.03802	0.70	2.51E-03	
11	Glucose	9.63	179.0551 (-)	59.01281, 71.01304, 85.02796, 101.02283	0.64	4.7502E-07	Glycolysis; Purine metabolism
12	Spermidine	11.44	146.1650 (+)	58.06551, 72.08084, 84.08069, 112.11225	0.64	1.4371E-08	Glutathione metabolism
13	GDP	9.72	442.0731 (-)	158.92393, 272.95660, 344,03995, 443.02112	0.64	1.79E-03	Purine metabolism
14	UMP	10.47	324.0827 (-)	78.95751, 111.01836, 150.98000, 192.99176	0.66	3.0614E-05	Nucleotide metabolism
15	GMP	10.28	362.0510 (-)	78.95744, 96.96821, 150.04089, 211.00046	0.70	5.21E-02	Purine metabolism
16	Acetylcarnitine	7.93	205.1228 (+)	60.08097, 85.02832, 144.10202, 145.04927	0.74	4.8379E-06	Insulin resistance
17	FAD	9.55	784.1515(-)	96.9680, 180.9899, 346.0565	0.77	3.60E-02	Riboflavin metabolism; TCA cycle
18	NAD+	10.13	662.1027 (-)	540.0539, 78.9573, 158.9237	0.78	7.70E-03	Oxidative phosphorylation, TCA cycle
19	NAG	9.42	188.0556 (-)	59.01289, 100.07552, 102.05466, 128.03409	0.79	8.69E-04	Arginine biosynthesis
20	Nicotinamide	1.42	123.0551 (+)	53.03894, 51.02333, 80.04942, 79.04166	0.81	4.20E-03	Nicotinate and nicotinamide metabolism
21	Methionine	9.49	150.0643 (+)	61.01081, 104.05289, 102.05496, 74.02349	0.81	9.89E-04	Cysteine and methionine metabolism
22	GDP-mannose	10.33	604.0705 (-)	78.95799, 150.04214, 158.92540, 320.97952	0.82	1.31E-02	Amino sugar and nucleotide sugar metabolism

23	NAA	9.94	174.0115 (-)	59.01282, 71.01286, 88.03890, 115.00228	0.84	1.02E-02	Alanine, aspartate and glutamate metabolism
24	cis-Aconitic acid	9.93	173.0080 (-)	85.02785, 111.00742, 129.01825	0.85	2.07E-02	TCA cycle
25	GSH	10.45	306.0568 (-)	128.03404, 160.00569, 210.08748, 254,07857	0.85	4.31E-02	Glutathione metabolism
26	Ornithine	10.41	133.0969 (+)	70.06522, 116.07065, 68.04974, 71.06849	0.87	4.32E-02	GSH metabolism
27	Histidine	10.01	156.0264 (+)	56.04977, 54.03417, 68.04957, 83.06026,	1.21	8.77E-03	Histidine metabolism
28	Pipecolinic acid	10.18	130.0862 (+)	56.04977, 67.05420, 84.08066, 55.05450	1.23	1.53E-02	Lysine degradation
29	Glutamine	10.36	147.0126 (+)	56.04977, 84.04429, 130.05026, 85.04743	1.24	4.94E-03	Glutathione metabolism; Purine metabolism
30	SAH	9.56	385.1286 (+)	88.02146, 97.02850, 136.06171, 250.07391	1.26	4.13E-03	Cysteine and methionine metabolism
31	SAMe	10.59	399.1440 (+)	97.02840, 136.06174, 250.09406, 298.09656	1.32	2.82E-03	Cysteine and methionine metabolism
32	Fumarate*	10.46	115.0022 (-)	68.9953, 71.0132	1.39	5.38E-02	Oxidative phosphorylation; TCA cycle
33	GTP	8.08	521.9955 (-)	424.00616, 344.03854, 272.95685, 158.93400	1.69	4.83E-02	Purine metabolism
34	AMP	10.11	347.0995 (+)	69.03358, 97.02832, 119.03513, 136.06168	1.69	4.81E-03	Purine metabolism
35	Cortisol	1.29	363.2161 (+)	121.06470, 309.18445, 327.19574, 97.06484	1.84	5.0711E-07	Cortisol synthesis and secretion
36	Glucose 6-P	10.35	259.0223 (-)	78.95747, 96.96814, 168.99004, 199.00037	2.85	8.60E-04	Amino sugar and nucleotide sugar metabolism
37	xylulose-5-P	10.04	230.0154 (-)	78.95746, 96.96814, 138.97887, 211.00240	3.60	2.49E-03	Pentose and glucuronate interconversions

* confirmed by commercial standards (confidence level: level 1).

Abbreviations: UDP-galactose, uridine diphosphate galactose; ASA, argininosuccinic acid; NAAG, N-Acetylaspartylglutamic acid; NADH, reduced nicotinamide adenine dinucleotide; GSSG, oxidized glutathione; UDP-GlcNAc, UDP-*N*-acetylglucosamine, GDP, Guanosine diphosphate; UMP, uridine monophosphate; GMP, guanosine monophosphate**;** NAD+, nicotinamide adenine dinucleotide; NAG, N-acetylglutamate; NAA, N-acetylaspartic acid; FAD, nicotinamide adenine dinucleotide; GSH, reduced glutathione; SAH, S-adenosylhomocysteine; SAMe, S-adenosyl methionine; GTP, guanosine triphosphate; AMP, adenosine monophosphate; Glucose 6-P, glucose 6-phosphate; xylulose-5-P, xylulose-5-phosphate.

REFERENCES

(1) van der Windt, G. J. W.; Chang, C.-H.; Pearce, E. L. Measuring bioenergetics in T cells using a Seahorse extracellular flux analyzer. *Curr. Protoc. Immunol.* **2016**, *113*, 3.16B.11-13.16B.14; DOI 10.1002/0471142735.im0316bs113.
